# Buscando Novos Parâmetros Não Invasivos para Prever a Fibrilação Atrial após Ablação por Cateter de Radiofrequência

**DOI:** 10.36660/abc.20230091

**Published:** 2023-03-20

**Authors:** Michael Ramon de Lima-Conceição, Jorge Lucas Teixeira-Fonseca, Danilo Roman-Campos

**Affiliations:** 1 Escola Paulista de Medicina Universidade Federal de São Paulo Departamento de Biofísica São Paulo SP Brasil Escola Paulista de Medicina, Universidade Federal de São Paulo – Laboratório de Cardiobiologia, Departamento de Biofísica, São Paulo, SP – Brasil

**Keywords:** Arritmias Cardíacas, Fibrilação Atrial, Ablação por Cateter, Eletrocardiografia/métodos

O Framingham Heart Study mostrou que a incidência e a prevalência de fibrilação atrial (FA) estão aumentando globalmente, com a prevalência aumentando mais de três vezes de 1958 a 2007.^1^ Em 2017, havia 37,57 milhões de casos prevalentes e 3,05 milhões de casos incidentes de FA globalmente , contribuindo para 287.241 óbitos.^2^ Notório que a estimativa mostra que esses números podem aumentar ainda mais no futuro. A projeção mostra que somente nos EUA, 12,1 milhões de pessoas até 2050 poderão ter FA.^3^ De acordo com a Diretriz da Sociedade Europeia de Cardiologia de 2020, a FA é definida como uma taquiarritmia supraventricular com ativação elétrica atrial descoordenada e contração atrial ineficaz. As características eletrocardiográficas da FA incluem intervalos RR irregularmente irregulares (quando a condução atrioventricular não está prejudicada), ausência de ondas P repetidas distintas e 3 ativações atriais irregulares.^4^ O diagnóstico clínico em pacientes sintomáticos ou assintomáticos com FA é feito por eletrocardiograma (ECG) de superfície, com duração mínima de 30 segundos de um ECG contendo traçado típico de FA.^5^

Embora exista uma definição clara do diagnóstico clínico de FA, distinguem-se pelo menos cinco padrões de FA, que se baseiam na apresentação, duração e término espontâneo dos episódios de FA.^4,5^ Muito provavelmente, a variedade de manifestações clínicas da FA está relacionada à etiologia da doença, que ainda não está totalmente elucidada. Existem vários preditores de desenvolvimento e progressão da FA, sendo os mais relevantes o índice de massa corporal, frequência cardíaca, idade, pressão arterial sistólica, história de hipertireoidismo, acidente vascular cerebral e insuficiência cardíaca.^6^ Devido à natureza complexa da FA, o manejo adequado do paciente é desafiador, o que implica, em uma situação ideal, um acordo coordenado e bem definido entre as vias de cuidado individualizadas do paciente para oferecer o tratamento mais adequado e otimizado. A abordagem de tratamento atual para pacientes com FA consiste na via holística A(trial fibrillation), B(etter), C(are), também conhecida como (ABC), que abrange: o ‘A’ Anticoagulação/Evitar AVC; ‘B’ Melhor manejo dos sintomas; e ‘C’ Otimização Cardiovascular e Comorbidade.^7^ É importante ressaltar que o tratamento também está sujeito a mudanças ao longo do tempo, principalmente pela descoberta de novos fatores de risco, progressão da doença, sintomas, ferramentas e métodos diagnósticos, preditores e desenvolvimento de novos tratamentos.

Na abordagem ABC, controlar o ritmo cardíaco é um passo fundamental para melhorar o controle do ritmo cardíaco, incluindo cardioversão, fármacos antiarritmicos e ablação por cateter.

Nesse cenário, alguns pacientes são refratários à terapia antiarrítmica farmacológica e, nesse caso, procedimentos minimamente invasivos estão se tornando cada vez mais comuns, como a ablação por cateter por radiofrequência (ACRF).^8^ O tratamento com ACRF busca interromper as vias elétricas anormais que causam batimentos irregulares.^9^No entanto, ainda não está claro se o ACRF, como primeira escolha de tratamento, está associado a melhores resultados clínicos.^10^ Uma metanálise recente de ensaios clínicos randomizados avaliou os benefícios da ACRF na manutenção do ritmo sinusal e na prevenção de arritmias refratárias, comparada com a terapia farmacológica.^10-12^ Um total de 24 estudos envolvendo 5.730 pacientes foram incluídos na meta-análise. A ablação por cateter reduziu hospitalizações, melhorou a fração de ejeção do ventrículo esquerdo e maior ausência de arritmia atrial em comparação com o tratamento farmacológico.^13^ Apesar da melhora na FA após ACRF, em alguns casos, o sucesso da ACRF não é claro. Assim, mais informações são necessárias para prever a eficácia da ablação de FA para orientar a seleção de pacientes apropriados e aumentar a taxa de benefício da ACRF. A esse respeito, nos Arquivos Brasileiros de Cardiologia^14^foi relatada uma metanálise avaliando a influência do volume do apêndice atrial esquerdo (VAAE) na recorrência de FA após ACRF. Os autores encontraram uma correlação significativa entre o VAAE e recorrência de FA após ACRF. Assim, os autores sugerem que o volume do apêndice atrial esquerdo pode ser um parâmetro confiável para determinar as condições estruturais e funcionais do átrio esquerdo em pacientes com FA inicial e usar essa abordagem para otimizar a terapia com ACRF.

Assim, futuros estudos de coorte são necessários para validar o valor preditor do VAAE e recorrência de FA após ACRF, o que acrescentaria novos parâmetros preditores não invasivos no manejo de pacientes com FA.
