# Supplementary material for: Buscando Novos Parâmetros Não Invasivos para Prever a Fibrilação Atrial após Ablação por Cateter de Radiofrequência
Source: Arq Bras Cardiol. 2023 Mar 20;120(3):e20230091. [Article in Portuguese] doi: 10.36660/abc.20230091 (PMC10392827; doi:10.36660/abc.20230091)
Supplement: Translation

# Translation: Chasing New Non-Invasive Parameters to Predict Atrial Fibrillation after Radiofrequency Catheter Ablation

**Authors:** Michael Ramon de Lima-Conceição, Jorge Lucas Teixeira-Fonseca, Danilo Roman-Campos

**Affiliations:** 1 Brazil Escola Paulista de Medicina, Universidade Federal de São Paulo – Laboratório de Cardiobiologia, Departamento de Biofísica, São Paulo, SP – Brazil

**Keywords:** Arrhythmias, Cardiac, Atrial Fibrillation, Catheter Ablation, Electrocardiography/methods

The Framingham Heart Study showed that the incidence and prevalence of atrial fibrillation (AF) are increasing globally, with the prevalence increasing more than 3-fold from 1958 to 2007.^1^ In 2017 there were 37.57 million prevalent cases and 3.05 million incident cases of AF globally, contributing to 287241 deaths.^2^ Notorious that the estimation shows that these numbers may further increase in the future. The projection shows that only in the USA, 12.1 million people by 2050 may have AF.^3^ According to the 2020 European Society of Cardiology Guideline, AF is defined as a supraventricular tachyarrhythmia with uncoordinated atrial electrical activation and ineffective atrial contraction. Electrocardiographic characteristics of AF include irregularly irregular R-R intervals (when atrioventricular conduction is not impaired), absence of distinct repeating P waves, and 3 irregular atrial activations.^4^ The clinical diagnosis in symptomatic or asymptomatic AF patients is performed by surface electrocardiogram (ECG), with a minimum 30 s duration of an ECG containing a typical AF tracing.^5^

Even though a clear definition of the clinical diagnostic of AF exists, however, at least five patterns of AF are distinguished, which are based on presentation, duration, and spontaneous termination of AF episodes.^4,5^ Most likely, the variety of clinical AF manifestations is related to the etiology of the disease, which is not fully elucidated yet. Several AF development and progression predictors exist, and the most relevant are body mass index, heart rate, age, systolic blood pressure, history of hyperthyroidism, stroke, and heart failure.^6^ Due to the complex nature of AF, proper patient management is challenging, which implies, in an ideal situation, a coordinated and well-defined agreement between patient-individualized care pathways to deliver the most adequate and optimized treatment. The current treatment approach for AF patients consists of the A(trial fibrillation), B(etter), C(are), a.k.a the (ABC) holistic pathway, which encompasses: the ‘A’ Anticoagulation/Avoid stroke; ‘B’ Better symptom management; and ‘C’ Cardiovascular and Comorbidity optimization.^7^ It is important to stress that the treatment is also subject to change over time, mostly because of the discovery of new risk factors, disease progression, symptoms, diagnostic tools and methods, predictors, and the development of new treatments.

In the ABC approach, managing heart rhythm is a key step to improving the control of heart rhythm, including cardioversion, antiarrhythmic medication, and catheter ablation.

In this scenario, some patients are refractory to pharmacological antiarrhythmic therapy, and in this case, minimally invasive procedures are becoming increasingly common, such as radiofrequency catheter ablation (RFCA).^8^ Treatment with RFCA seeks to disrupt the abnormal electrical pathways that cause irregular beats.^9^ However, it is still unclear whether RFCA, as the first treatment choice, is associated with better clinical outcomes.^10^ A recent meta-analysis of randomized clinical trials evaluated the benefits of RFCA in maintaining sinus rhythm and preventing refractory arrhythmias compared with pharmacological therapy.^10-12^ A total of 24 studies involving 5,730 patients were included in the meta-analysis. Catheter ablation reduced hospitalizations, improved left ventricular ejection fraction, and greater absence of atrial arrhythmia compared with drug treatment.^13^ Despite the improvement in AF following RFCA, in some cases, the success of RFCA is unclear. Thus, further information is necessary to predict the effectiveness of AF ablation to guide the selection of appropriate patients and increase the benefit ratio of RFCA. In this regard, in the of *Arquivos Brasileiros de Cardiologia*^14^ it was reported a meta-analysis evaluation of the influence of left atrial appendage volume (LAAV) on the recurrence of AF following RFCA. The authors found a significant correlation between LAAV and AF recurrence after RFCA. Thus, the authors suggest that the left atrial appendage volume could be a reliable parameter for determining the left atrial structural and functional conditions in early AF patients and use such an approach to optimize RFCA therapy.

Thus, future Cohort studies are needed to validate the predictor value of LAAV and recurrence of AF after RFCA, which would add new no-invasive predictor parameters in the management of AF patients.
